# On-orbit sleep problems of astronauts and countermeasures

**DOI:** 10.1186/s40779-018-0165-6

**Published:** 2018-05-30

**Authors:** Bin Wu, Yue Wang, Xiaorui Wu, Dong Liu, Dong Xu, Fei Wang

**Affiliations:** State Key Laboratory of Space Medicine Fundamentals and Application, China Astronaut Research and Training Center, No. 26 Beiqing Road, Haidian District, Beijing, 100094 People’s Republic of China

**Keywords:** Astronaut, On-orbit, Sleep, Countermeasure, Human spaceflight

## Abstract

Sufficient sleep duration and good sleep quality are crucial to ensure normal physical and mental health, cognition and work performance for the common people, as well as astronauts. On-orbit sleep problem is very common among astronauts and has potential detrimental influences on the health of crewmembers and the safety of flight missions. Sleep in space is becoming a new medical research frontier. In this review we summarized on-orbit sleep problems of astronauts and six kinds of causes, and we presented the effects of lack of sleep on performance as well as mental and physical health, then we proposed seven kinds of countermeasures for sleep disturbance in spaceflight, including pharmacologic interventions, light treatment, crew selection and training, Traditional Chinese Medicine and so on. Furthermore, we discussed and oriented the prospect of researches on sleep in space.

## Background

It is well known that good sleep is very important for keeping normal physical and mental health, cognition and work performance for common people. Good sleep generally includes sufficient sleep duration and good sleep quality. Unfortunately, evidence has consistently shown that disrupted sleep is a very common and important problem among astronauts [1, 2]. In human spaceflight, sleep duration and sleep quality of astronauts were adversely affected by combined special factors including microgravity, isolation, monotonous repetition, high vigilance workload and so on. Sleep problems could impair the work performance and health of crewmembers which could ultimately influence the safety of flight missions. NASA (National Aeronautics and Space Administration) has listed sleep deprivation and circadian rhythm changes as important risk factors during long-term flight [2]. On the basis of several times of short-term manned spaceflight practices, China has attached more and more importance to on-orbit sleep problems of astronauts and considered it as one of the key factors for keeping human performance capabilities in medium and long-term spaceflight [3]. In recent years, researchers focus on the effects of space flight on sleep and the countermeasures which gradually become a new frontier of space medicine [1–4]. In this article, the research progress in this field was reviewed and analyzed to provide reference for the study of sleep medicine in medium and long-term human spaceflights, so as the circumstances for similar extreme environmental practitioners in China.

### On-orbit sleep problems of astronauts and causes

#### Sleep time

Unlike other medical problems in human spaceflight, sleep in space did not attracted much attention until 1976. For the first time, Kanas et al. [4] and Frost et al. [5] reported the sleep condition of three American Skylab astronauts by polysomnographic analysis. The results presented that the daily sleep time on orbit was only 6 h on average which was 1 h less than on the ground. In 1988, Santy et al. [6] found that 58 shuttle astronauts slept for an average of 6 h each night during spaceflight with the comparison of 7.9 h sleep time on the ground. Many of them reported less than 5 h sleep on some nights, even less than 2 h. It should be mentioned that scheduled rest-activity cycles were 20–35 min shorter than 24 h in shuttle missions. In 2014, Barger reported that 64 shuttle astronauts and 21 International Space Station (ISS) astronauts slept 6 h and 6.1 h respectively on average each night through the analysis of the actigraphy data and the flight log. Further analysis showed that the crewmembers of shuttle missions slept 20 min shorter than the pre-launching period (2 weeks), and 47 min shorter compared with the post-landing period (1 week), while ISS astronauts slept 19 min longer than that in 2 weeks before the flight each day, and 52 min shorter compared with the 1 week after landing. They believed that due to preparations of the flight missions and other workloads, the astronauts had already lacked of sleep 2 weeks before the flight [7].

In 2015, NASA summarized the data of 177 astronauts from 7 studies including the study above and found that researches in different years, by different researchers or with different methods went to a consistent result that astronauts had about 6 h daily sleep time on average during spaceflight [2]. Obviously, on-orbit sleep time of the astronauts was significantly less than the time recommended by National Sleep Foundation and the American Academy of Sleep Medicine to maintain ideal alert performance and health [8, 9]. It was 2 h less than the 8 h specified in the NASA-STD-3001 (Vol.1) [10].

In Shenzhou-9 and -10 missions of China, the average daily on-orbit sleep time of 6 astronauts was generally less than 8 h, which was arranged in advance. Especially in the early days of flights, sometimes astronauts slept less than 5 h daily (data not shown).

#### Sleep quality

With sleep recorder, some objective studies showed that the sleep structure of the astronaut has changed during spaceflight. Gundel et al. [11] studied the sleep of astronauts on the Mir space station and found that the latent period of the first rapid eye movement (REM) phase was short, and slow-wave sleep (SWS) was redistributed between the first and the second sleep cycles. They also reported that one astronaut had extended sleep latency and poor sleep efficiency, which was defined as “space insomnia”. A study on the American astronauts of Mir mission found that REM sleep time in the space was reduced by 50% compared to that of preflight. Although the longer bed rest during the flight, the overall sleep time was 27% less than that before the flight [2, 12]. Moldofsky et al. [13] studied the EEG of eight astronauts in the Mir space station and found that SWS time was significantly less than that before the flight. Dijk et al. [14] reported that 5 astronauts performing the short shuttle flight tasks, during the last third period of the flight, had increased waking time and decreased SWS, and REM rebounded significantly after the flight.

Contrary to most objective studies reported that sleep quality got worse in flight, results of subjective findings were inconsistent with the objective ones. Barger et al. [7] and Dijk et al. [14] reported that compared with the sleep before the flight, the astronauts gave a “bad” subjective evaluation of the quality of in-flight sleep. Some Chinese astronauts, who have flown short-duration space missions, occasionally complained that their sleep became easily affected (data not shown). But Dinges et al. [15] studied sleep logs and found that astronauts rated their overall orbital sleep quality at “good” grades. Whitmire et al. [16] also reported their interviews and surveys of astronauts after the flight, of 52% saying that they had better sleep during flight, and only 6% with the reverse feedback. Since these evaluations were retrospective, the reliability could be questionable. Based on the analysis of ISS astronauts’ diary, Stuster et al. [17] found that astronauts felt much tired in the first quarter of flight compared to the late phase of flight, which was related to the decreased sleep quality.

#### Other evidences of sleep disturbance in spaceflight

Other compelling evidences were the reports of sleep medication used in flight by astronauts. In 1988, Santy et al. [6] reported that 50% (11/22) of shuttle crewmembers on dual-shift missions used sleep medications at least once inflight compared to 19.4% (7/36) of single-shift. In 1999, NASA reviewed 219 records (each record represented one crewmember) from 79 shuttle missions, 94% of crewmembers had used medications, and 45% of crewmembers using drugs to solve the sleep disorder [18]. In 2014, Barger reported 78% (61/78) of the crews of space shuttle missions in 52% (500/963) of the nights took a dose of drugs to promote sleep, in the night of 17% (87/500) took twice to promote sleep, and 75% (12/16) of individuals on ISS had used drugs to promote sleep. A total of 852 sleep logs were collected, 96 reports had mentioned using sleep promoting drugs, and 18 reports had mentioned using sleeping pills twice [7]. Further statistical analysis showed that sleeping problems, space motion sickness, and pain remained the top 3 complaints among astronauts. And the two most frequently used drugs were sleep medicine and the drug for rash, and the use of sleeping pills was at least 10 times more than normal Americans on average [2, 18–21].

In addition, Stuster et al. [17] analyzed the astronaut diaries with the evidence of sleep inertia existing during spaceflight, that is, astronauts could not quickly switch from sleep to wakefulness. These evidences support the apparent adverse effects of spaceflight on sleep.

#### Causes of sleep problems in spaceflight

Studies demonstrated that some of the astronauts’ sleep problems in space were often caused by uncomfortable ambient temperatures, higher noise levels, uncomfortable sleeping bags, or the absence of familiar proprioceptive cues [11, 22].

During the flight, when sleep time was suddenly interrupted by operational needs or social activities, astronauts were prone to sleepless and fatigue. According to reports, there was a sudden change of schedule in 13% of 2043 days aboard ISS, and usually occurring during or before the key operation (such as spacecraft docking or detachment, extravehicular activity etc.) [23]. Sleep would be also affected by the suddenly shifted operations, tasks arranged at night or incomplete schedule due to high workload, etc. Some astronauts believed that the unreasonable workload arrangement was the main reason of the poor sleep quality and the shorter sleep time in flight [16, 17].

Sleep changes may be related to impaired sleep homostatic regulation induced by space environment. According to the accepted model, two interacting processes are involved in this regulation, and the first is called the “S process”. It represents a homeostatic process that is reflected in an increase of sleep propensity over the waking phase and a decline of this propensity during sleep. A direct physiological marker of this process is the portion of slow wave activity in the human EEG [24]. SWS reduction could be seen as a sign of changes of “S process” in space [4].

The absence of circadian cues, including light cycles or even attenuated light, seems to disrupt human biological rhythms [14, 25]. Some studies have found that, compared with the ground control, some physiological parameters related to the circadian rhythm, such as body temperature and cortisol, decreased or lagged in phase for the astronauts in space [11, 14, 26, 27]. In 2015, Flynn-Evans et al. [28] applied a mathematical model to estimate the timing of the circadian nadir among 21 astronauts aboard ISS; crewmembers were studied an average of 155 days each, 3248 days total. It was reported that the estimated circadian phase occurred outside the sleep episode 19% of the time during the spaceflight. It can be seen that circadian rhythm disorder may be an important cause of orbital sleep disturbance to astronauts.

In addition, human spaceflight practice had proved that astronauts experiencing a long-term spaceflight suffered more prominently from psychological and physiological problems. The adverse psychological reaction includes depression, anxiety, personality changes, and intra-crew conflicts, the adverse physiological reaction includes cardiovascular deconditioning, muscle atrophy and decrease of immune response and so on, which would inevitably lead to some functional or organic disorders and diseases, such as space adaptation syndrome, pain, infection and eye disease and so on. All those stress factors could cause sleep problems, which in turn could increase psychological/physical discomfort [4, 29].

### Effects of lack of sleep on performance and mental and physical health

Many ground investigations showed that sleep insufficiency would affect human physical and mental health, and induce performance degradation [30–33]. A report on the US Challenger space shuttle accident posited that lack of sleep and irregular work schedule were important reasons for senior managers making critical decision error [34].

Up to now, there is few research on cognitive performance of human in spaceflight, especially related to sleep insufficiency. In the retrieved eight tests on orbit, five tests showed that there was a negative effect on cognitive performance including attentive search, short-term memory, tracking operation, careful operation or dual task, two tests showed no effect, and one showed a slightly positive effect [2, 14, 27, 35–38]. Dijk et al. [14] analyzed the relationship between the cognitive performance and sleep in flight and found that the performance of most astronauts declined one week before the launch, with a further reduction in flight and slow recovery after flight. Both performance degradation in flight and performance improvement post flight were related to REM sleep. Nechaev et al. analyzed the error data of 28 astronauts and 342 weeks of 14 missions on the Mir space station and found that the error rate was significantly related to the deviation degree of the normal sleep-wake cycle [39].

Compared with the relatively scarce space tests, a large number of studies based on ground simulations, which focused on the effects of sleep insufficiency, have been carried out [40–44]. These results showed that sleep less than 6 h during two consecutive nights could negatively affect cognitive performance, such as decreased response time, increased error, impaired working memory, and so on. Moreover, the impaired performance would last within 1 week. If this condition was further extended, negative effects on cognitive performance would be gradually accumulated which showed a much obvious dose-time effectiveness relationship [40]. Furthermore, the effect of chronic sleep deprivation on performance was as similar as acute total sleep deprivation. Van Dongen et al. [40] reported that 4–6 h of sleep for 14 consecutive nights was equivalent to 48 h or 24 h of sleep deprivation. Belenky et al. [41] found that sleeping for less than 6 h in 7 consecutive nights caused impaired performance, hardly returned to normal levels, even after 3 nights of sleep free.

In addition to performance impairment, other negative psychological and physiological health problems caused by lack of sleep have also attracted researchers. In 2009, NASA reported that 36 h of sleep deprivation deteriorated the emotional state of the participants including energy level, arousal state, motivation and concentration [45]. In 2010, Van der Helm et al. [46] found that 30 h sleep deprivation resulted in that participants were unable to correctly identify two types of facial expressions, anger and pleasure. This may be an important reason for the social relation problems of crews in the long-term spaceflight. In 2014, Minkel et al. [47] reported that one night of sleep deprivation could lead to the level of cortisol in a quiet state, and further increase when participants accepted social stress, and it may adversely affect the long-dated health.

Considering astronauts’ living environment including confinement, isolation, and other factors, some scholars have carried out researches on the effects of sleep deprivation combined with restrictions. Chaumet et al. [48] found that the tendency of adventure decreased in the 36 h sleep deprivation, while the impulsiveness of that increased with normal sleep in the closed environment. Spitznagel et al. [49] reported that sleep deprivation and cold exposure for 53 h had a superimposed effect on performance impairment. NASA had completed a 45 d confined experiment named “Hera” experiment with four participants on July, 2017. During Hera, all crews were required to sleep 5 h per night of 1 week, in the rest 2 days of which they could sleep 8 h for recovery. The purpose of this study was to investigate the effects of countermeasures on sleep deprivation under simulated spaceflight isolation [50]. The results of this research have not been reported yet.

Since 2006, our team has carried out a series of studies about the effects of 72 h sleep deprivation on human physiology, psychology, performance and countermeasures in confined and isolated environment. Although the situation of 72 h sleep deprivation in space had never been reported yet, the effect of sleeping 6 h per night in consecutive beyond 2 months might be analogical. With the consideration of further long-term spaceflight, for example, the Mars mission could be more than 1 year, the situation of 72 h sleep deprivation could not be entirely ruled out. Furthermore, we could investigate the effects of 24 h, 36 h and 48 h sleep deprivation within 72 h schedule. It was also important for the success of the current spaceflight missions. The results of the research on the countermeasures will be briefly described in the next section. The following are the main results of our previous works:

#### Effects on cognition

The confined and isolated environment for 72 h with or without sleep deprivation slowed perception, and the effect of sleep deprivation was greater, but the perceived accuracy was not affected [51]. Simple isolation for 72 h had no significant effect on working memory, prospective memory and attention network. But in the late period of the test which was 72 h confined isolation with sleep deprivation, the reaction time was prolonged, the rate of accuracy decreased and the rate of leakage increased. The functional nuclear magnetic resonance imaging (fMRI) studies showed that for attentional tasks, confined isolation with sleep deprivation diminished the endogenous attentional system in the brain and decreased brain top-down control, while the stimulus driven attention system was enhanced [52–55].

#### Effects on visual alertness

Continuous performance task test revealed that 72 h confinement and isolation with sleep deprivation resulted in a significant decrease in visual alertness. The fMRI showed that, compared with pre-experiment, the volunteers’ thalamic gray matter volume was significantly reduced, but the volume of hippocampus and the brain gray matter did not change after the experiment, indicating that the decrease in visual alertness was associated with a decrease in gray matter volume in the thalamus [56].

#### Effects on operational performance

We found that 72 h sleep deprivation with isolation had negative effects on 3 levels of complexity and 3 types of simulated space emergency operation performance. However, highly complex operations and manual type operation performances remained relatively stable in our experiment, whereas low complex operations and two-way discrimination type operations were significantly affected [57]. This is similar to some previous reports, and generally engaging in challenging and stimulating tasks can be used to compensate for the effects of sleep deprivation stress through the compensatory mechanism of the brain [30, 58, 59]. Although we also found that for the difficult manual rendezvous and docking operation, the unitary isolation condition had no adverse effect on the volunteers’ performance. However, the isolation coupled with sleep deprivation could significantly reduce the docking success rate. The docking fuel consumption, displacement deviation, pitch and yaw angle deviation increased significantly, which seriously weakened the operation performance of volunteers [54]. This suggested that sleep deprivation have a complex impact on space performance, and the characteristics of the operation itself, such as the need of relying on the attention network, may be a very important factor.

#### Effects on emotion

Chinese version of the State-Trait Anxiety Inventory, the questionnaire Self-Rating Depression Scale, the Positive and Negative Affect Scale, and a brief Profile of Mood States Cale (POMS) on mood changes were used in our study on a laptop. We found that 72 h isolation had no obvious effect on individual emotion. But exposed to an isolation environment with 72 h sleep deprivation, anxiety score and POMS score of emotional confusion of volunteers increased significantly in the latter phase of the experiment while depression scores did not change significantly. Notably, there was no significant change in the negative mood scores of the volunteers with 72 h isolation and sleep deprivation, while the positive mood score significantly decreased. The results above indicated that the psychological support strategy to promote the positive mood of astronauts might be a good method to deal with the sleep insufficiency in space [52, 53, 60].

#### Effects on reaction to emotional faces pictures

Before and after 72 h confined isolation with sleep deprivation test, volunteers were arranged to browse emotional faces pictures. The fMRI was performed after isolation. The results showed that when volunteers viewed low intensity anger pictures after the experiment, they showed activation of brain areas the same as high intensity anger pictures, intimating that the threshold of negative emotion was decreased and the sensitivity was increased. The findings were similar to the findings by van der Helm, further speculated that the lack of sleep may be an important reason for the tense relationship among members of the group during long-term spaceflight [46].

#### Effects on physiological and biochemical parameters

During the period of 72 h isolation and sleep deprivation, the heart rate and body temperature of volunteers showed a trend of high in the day and low at night, and the circadian rhythm of systolic and diastolic blood pressure was not obvious. After the experiment, the levels of growth hormone, cortisol, and dopamine in serum significantly increased, while melatonin slightly decreased, compared with pre-experiment. These results suggested that closed isolation with sleep deprivation affects the biological rhythms of volunteers and increases their excitability (data not shown). The changes of cortisol of blood in the experiment were similar to those of Minkel et al. [47].

### Countermeasures for sleep disturbance in spaceflight

#### Improve sleep environment

Creating a good environment in the space cabin is beneficial to ensure the sleep quality and sleep time thus to meet the performance and health needs of the crew. Comparing with the relatively primitive sleep conditions in short-term shuttle flights, ISS has improved the habitability in ambient temperature, wind speed, noise, and carbon dioxide levels in addition to comfortable sleeping bags, restraints to prevent floating, and private sleep quarters to minimize interruptions. This process is continuously developed with obvious progress. However, much more efforts need to be invested, especially in the noise control [2, 11, 27, 61, 62].

#### Design reasonable on-orbit work-rest schedules

A detailed on-orbit work-rest schedule should be designed according to launching and landing time, crew numbers, time critical events, load demands, mission objectives, and shift mode before the flight mission. NASA recommended that astronauts sleep 8 h or at least 6 h every day on orbit. After work shift, sleep time can be prolonged by 1.5 h than the day before. Furthermore, interesting works, enough time for rest and recreation should be arranged in the schedule to avoid sustained fatigue [2].

Stuster et al. [17] reported that astronauts on ISS were more inclined to take naps in daytime to improve their sleep quality in the middle and late stages of the flight missions. Some ground-based studies also supported the viewpoint that fragmentary sleep or short nap in daytime might contribute to alleviate the impairments of performance caused by sleep deprivation at night [63, 64]. But after observing and analyzing the situation of a volunteer in Mars 500 simulation experiment, Basner et al. [65] found that self-selected naps in daytime may cause biological rhythm disorders during long-term missions and thus should be arranged and monitored carefully. It deserves further discussion that whether taking naps in daytime could be a recommended countermeasure or not.

Some other ground-based studies indicated that it may be helpful to have sleep extension before or after sleep restriction. Banks et al. [66] carried out an experiment, in which 159 participants had been given 0–10 h recovery sleep after undergoing 4 h of nocturnal time in bed every night for 5 consecutive days. They found that neurobehavioral functions were improved with recovery sleep time and have obvious quantity-effect relationship. By observing the task performance of 24 subjects who had underwent seven sleep restriction nights (3 h in bed) after seven adequate sleep nights (7–10 h in bed), Rupp et al. [67] found that although sleep extension before sleep restriction didn’t affect reaction time, it improved cognitive function.

Our research indicated that reasonable collocation and time assignment of different complexities and types of tasks could be used in designing work-rest schedule to prevent the negative effects of sleep deprivation on performance [57]. The astronauts of Shenzhou-9 and -10 missions in China suggested that sleep time arrangement on orbit should be personalized to a certain extent and giving astronauts some autonomy could improve sleep efficacy. Currently, NASA is studying how to modify flight schedules to allow for adequate sleep and time off on a case-by-case basis. At the same time, a scheduling dashboard is under development to track behavioral health and mission stressors and to enable early stage detection and mitigation [2, 68]. The schedules with new countermeasures will be verified and objectively evaluated in future spaceflight.

#### Pharmacologic interventions

Sleep medication is the most prevalent countermeasure during spaceflight. Barger et al. [7] and Whitmire et al. [16] reported that more than 70% of both shuttle and ISS astronauts use sleep medications during the flight missions, which hastened the approach of sleep but did not bring longer sleep duration. Dijk et al. [14] conducted a rigorous controlled trial during space shuttle flights. They found that melatonin significantly improved sleep latency compared to placebo, but there was no difference in other sleep parameters. The sleep medications frequently used in space include zolpidem, zaleplon, continuous release zolpidem, flurazepam etc. The sleep medications occasionally used include temazepam, eszopiclone, melatonin, quetiapine fumarate etc. [7, 16, 61]. Three sleep medications were provided on orbit in China space laboratory mission. They were triazolam, zolpidem and diphenhydramine, in which diphenhydramine was also effective for motion sickness.

Numerous ground-based studies on shift work staff demonstrated that zolpidem improved sleep quality and increased sleep duration, but decreased mood on the following day, which requires attention [69, 70]. Unlike other sleep-inducing hypnotics, melatonin is used primarily to shift the circadian rhythm. Some studies showed that melatonin modestly improved sleep efficiency during circadian misaligned sleep episodes relative to placebo [71, 72]. By animal experiments, Wang et al. [73] from China Astronaut Research and Training Center has proved that midazolam nasal gel spray possesses obvious sedative and hypnotic effects and by nasal administration it is easy to use with rapid effect and high bioavailability. Midazolam nasal gel spray is expected to be used in the flight because of its shorter T max and higher bioavailability.

Stimulant could be used in flight when an astronaut needs to overcome drowsiness and stay awake. In the records of Apollo 7 mission, the sleep condition of an astronaut was too bad that he fell asleep on duty and had to take 5 mg amphetamine to stay awake. In post-flight interviews, Whitmire et al. [16] found that 75% of the astronauts had used caffeine or modafinil as stimulants during their missions. In China space laboratory mission, caffeine was also provided on orbit.

Ground-based researches indicated that caffeine improved the decline of alert, cognition and operation performance caused by sleep deprivation, particularly for emergent situations where extended wakefulness or where a rapid transition from sleep to wake is required [74, 75]. By comparing the effectiveness of caffeine, modafinil and dextroamphetaminein different sleep deprivation trials, Killgore et al. [76, 77] found that all the three stimulants significantly improved performance compared to placebo. In this case although caffeine resulted the fastest, it was associated with the most negative side-effect. Dextroamphetamine had the longest latency to improve and caused disrupted recovery sleep. Modafinil had no side effects. In 2017, Chinese researchers reported that doses of Ginsenoside Rh1 could prevent cognitive impairment caused by sleep deprivation in mice, and the effect was similar to that of modafinil [78]. This suggested that some active ingredients of Traditional Chinese Medicine (TCM) are potential irritants with further exploration value.

More work needs to be done to study the efficacy, side effects, and method of administration of sleeping medication on orbit, so as to screen and develop individualized sleep medications and stimulants with high safety.

#### Light treatment

As an effective stimulus to regulate human circadian rhythm, neuroendocrine and neurobehavioral response, light could be used to treat sleep disorders and maintain health of individuals in intercontinental flight, shift work and spaceflight [79, 80].

The human circadian pacemaker is most sensitive to short-wavelength blue light ranging from 460 to 480 nm [81]. Through human experiment, West et al. [82] demonstrated that the suppression of lower intensity blue light to melatonin is greater compared with broad spectrum bright light at night, and that blue irradiances above 20 μW/cm^2^ significantly suppressed melatonin in a dose-response manner, with higher irradiances eliciting greater suppression. Given that melatonin suppression is associated with improved alertness and performance, a study by Rahman et al. [83] demonstrates that lower intensity blue light has better feasibility than the broad spectrum bright light.

A research manifested that the human visual system had peak sensitivity to green light of approximately 555 nm. Green light is capable of eliciting melatonin suppression, which is similar to blue light, but the effect of green light is temporary [84]. These findings suggested that it may be possible to use a combination of green light and blue light to optimize light-induced circadian phase shifting. Zeitzer et al. [85] found that light flashes of 2 milliseconds given every 30 s were sufficient to cause a phase shift of approximately 30 min. It means that using millisecond flashes of light to promote sleep or wakefulness in space could reduce energy consumption.

In 1990, on NASA’s Space Transport System-35 mission, crewmembers were exposed to timed, bright white fluorescent light at 10,000 lx during their preflight, quarantine period at Johnson Space Center. This intervention successfully realigned crewmembers’ melatonin rhythm with their required sleep-wake cycle [86]. Subsequent space shuttle missions employed the program that included a preflight light therapy regimen. In the Payload Operations Control Center at Marshall Space Center, a study was done testing the utility of bright light treatment of NASA ground crew who worked on shifted schedules. 18 ground crew personnel were divided into an experimental group and received schedules for bright white fluorescent light exposure at 10,000 lx, with sunlight avoidance. The control study participants underwent no treatment. The results showed that the experimental group had better sleep, performance, and physical and emotional well-being [87]. During the Phoenix Mars Lander mission, NASA conducted a research to test the effectiveness of a lighting countermeasure to synchronize the circadian system of operational ground personnel supporting the 3-month mission, living on a Mars sol (24.6 h) at mission control. A portable light box containing arrays of blue LEDs was placed on the desk of the participants to provide a photic time cue to facilitate circadian adjustment to the longer day length. The circadian rhythm of the urinary metabolite of melatonin, 6-sulphatoxymelatonin (aMT6s), was used to assess circadian period. The results demonstrated that 87% of participants were able to adapt to the Mars sol [88]. It seems that lighting countermeasures are necessary for both astronauts and ground crew in long-term spaceflight missions.

The lighting facilities on board ISS had been changed from General Luminaire Assemblies to Solid-State Lighting Assembly (SSLA) at the end of 2016. This was an important step in the development of light therapy from ground research into space practice. Three different LEDs were used for the ISS SSLAs: a broadband LED composed of a highly converted green phosphor that emitted white light, a blue LED that emitted narrow band blue-appearing light with a peak emission of 468 nm, and a red LED with a peak emission near 626 nm. This new lighting system provides three settings: general illumination mode, phase shift/alertness mode, and pre-sleep mode. Based on operational tasks being performed or crewmember preference, further lighting control was possible in each setting via a dimmer switch to meet the visual demand and as lighting countermeasures against sleep disorder and circadian rhythm disorder in ISS [62, 80]. We look forward to new encouraging research achievements to be reported.

#### Psychological support

Appropriate psychological support or intervention could be helpful for astronauts to cope with the problems in falling asleep and poor sleep quality, and it has good effects on relieving fatigue, promoting rest and sleep [61, 62, 89]. With measures such as sleep cognitive behavioral intervention, direct or indirect psychological support to the astronauts had good effects, which started from space station missions of the US and Russia and had been used in Shenzhou-9, − 10 and − 11 missions. Before Mars 500 experiment, training of mood regulation skills, sleep-promoting skills and leisure time management consciousness were conducted on the Chinese volunteer by our team. During Mars 500 experiment, the volunteer made psychological self-adjustment and received professional psychological support. All the measures helped the volunteer greatly to maintain positive mood and to deal with sleep issues (unpublished information). In addition, in the 72 h sleep deprivation experiment, our team found that comprehensive psychological intervention including improving self-confidence, active resource integration and so on, which can effectively reduce the negative effect of sleep deprivation on mood state and alleviate the decline of operation performance [90].

#### Crew selection and training

Due to individual difference as well as the great potential and plasticity in physiology and psychology, the selection and training of crew can be taken as an important measure to cope with the sleep issues in spaceflight.

In the 72 h sleep deprivation experiment, our team found that a few volunteers experienced significant cognitive and performance decrements and extreme bad emotion (unpublished information). Rupp et al. [91] found that the subjects with poor PVT performance and emotional display were more likely to be affected by sleep restriction. Recent studies have found that certain genetic polymorphisms are associated with vulnerability to sleep loss. Groeger et al. [92] and Vandewalle et al. [93] reported that the subjects with *PER3*^*5/5*^ genotype variant had poorer ability to complete tasks and had vulnerability in response to sleep deprivation and rhythm disorder compared with those carrying the *PER3*^*4/4*^ variant. An et al. reported that the main genotype of *PER3* in Han Chinese is *PER3*^*4/4*^. The genotype frequency of Han Chinese was 78.33% for *PER3*^*4/4*^ (twice the frequency of Caucasians, African Americans and Italian), 1.67% for *PER3*^*5/5*^(1/10–1/6 the frequency of Caucasians, African Americans and Italian) [94]. In addition, Goel et al. [95] found that the COMT Val158Met polymorphism may be a genetic marker for predicting individual differences in sleep homeostasis. These results suggest that the sleep deprivation experiment and genotype identification can be used to screen those of vulnerability in response to sleep deprivation and rhythm disorder, so as to ensure the astronauts who carry out missions on orbit have better adaptability to environment and assignment. Further research is necessary in this field.

Around 2013, our team conducted training for some Chinese astronauts on adaptability to sleep deprivation in a closed isolation environment for 72 h. The astronauts were divided into three groups and were given space food during the period. They completed a number of tests and simulated space operations. In 2015, the adaptability training was carried out for 6 oceanauts on “Jiaolong” manned deep-sea submersible for 36 h by our team. Good effects had been achieved from the training, mainly reflected in three aspects. The first, emotion, cognition and operation performance of the trainees were significantly better than that of ordinary healthy volunteers, which showed the role of selection and training. Secondly, the characteristics of the psychological and physiological responses of each trainee in the special environment were recorded and will provide important basis for specific psychological support and training and medical safety in the future. Thirdly, the trainees had fully experienced the reactions in the special environment, mastered the coping methods and improved their tolerance (unpublished information).

#### TCM and other measures

**Fig. 1:**
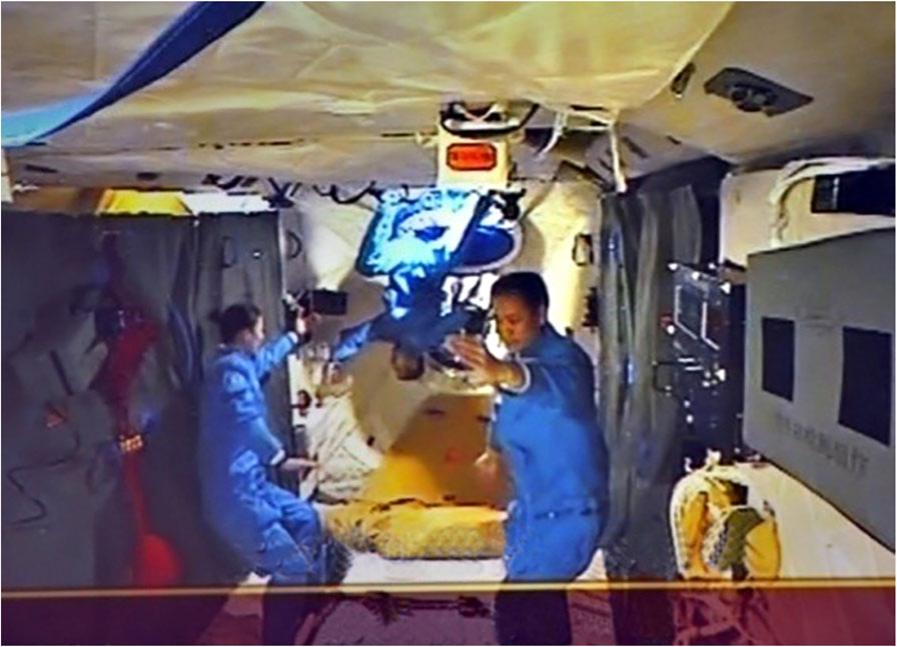
The practice of Tai Chi during Shenzhou-10 mission

In the experiment of sleep deprivation in narrow and sealed environment, our team studied the effect of Tai Chi training on mood and EEG spectrum power. The study showed that Tai Chi training could improve mood and reduce the low frequency activity of the EEG signals, indicating that this training has obvious antagonism on sleep deprivation [96]. We speculate that Tai Chi training also helps to hasten sleep and keep deep sleep. In Shenzhou-10 and -11 missions of China, some astronauts practiced Tai chi and initially proved its feasibility in microgravity (Fig. 1). The effects of Tai Chi as a potential countermeasure for sleep problems need to be seriously investigated and proved.

The methods commonly used in TCM, such as acupoint stimulation, acupuncture and massage, as well as transcranial magnetic/electric stimulation and eating food which helps for sleep or wakefulness, were proved to be effective on promoting sleep, staying alert or improving cognition in the ground-based research [97–99]. These potential methods are expected to be further evaluated and possibly applied in Chinese space station.

## Summary and prospect

**Fig. 2:**
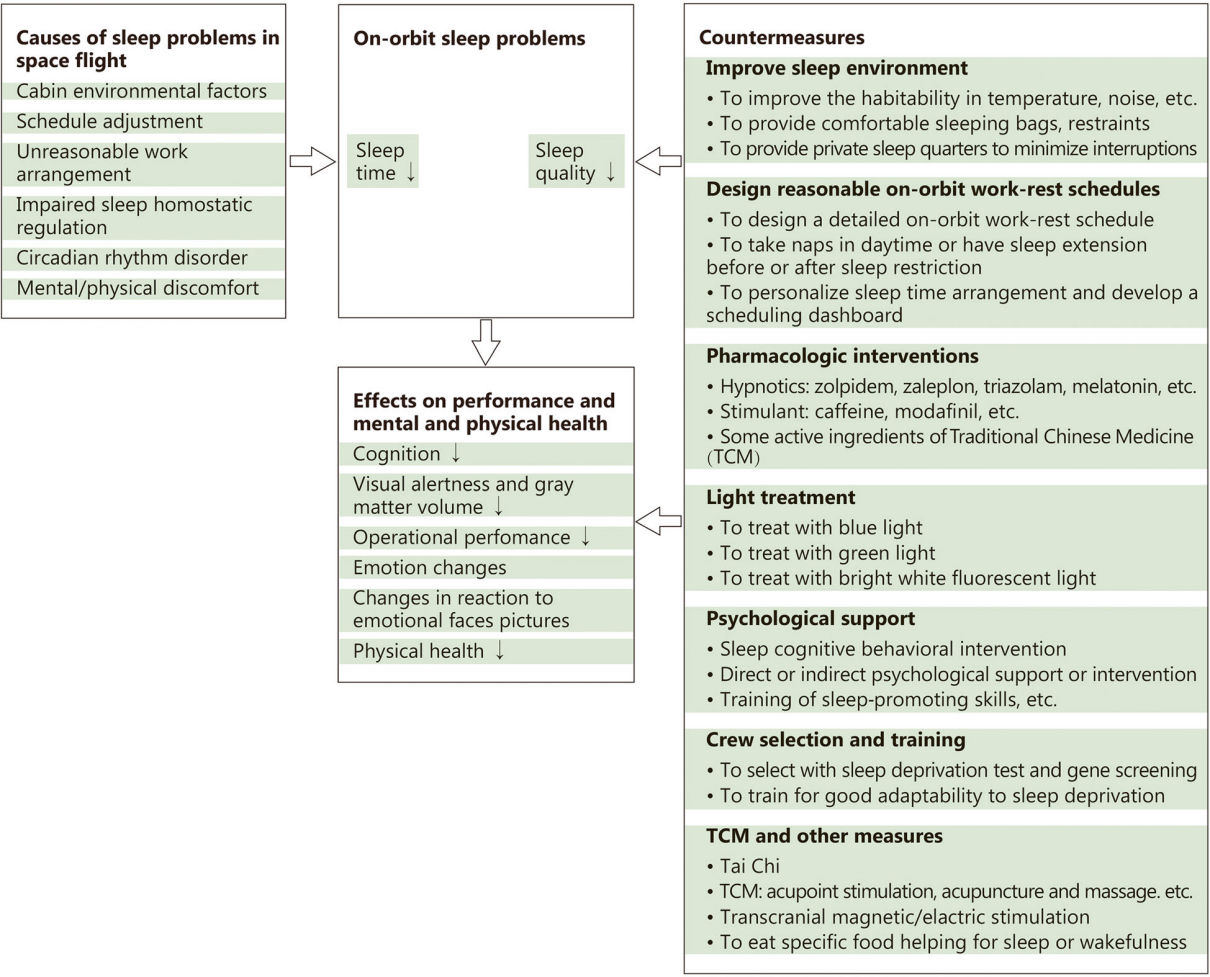
Frame diagram of on-orbit sleep problems of astronauts and countermeasures. In human space flight, sleep time and quality on orbit are apparently decreased. This figure presents six kinds of causes, some effects of sleep deficiency on performance and mental and physical health, and seven kinds of countermeasures for sleep disturbance in spaceflight. The symbol of ‘↓’ means decreased or impaired

We summarized on-orbit sleep problem of astronauts and countermeasures as Fig. 2.

In spaceflight, sleep insufficiency and worse sleep quality are common problems for astronauts. The main causes of sleep disturbance in spaceflight include the confined cabin, the adjustment of work-rest schedule, the unreasonable working arrangement, the impaired sleep homostatic regulation, the circadian rhythm desynchrony, the psychological/physical discomfort, etc. Fewer on-orbit studies about the effects of sleep on behavioral performance and lots of ground-based studies on the effects of sleep deprivation (restriction) indicated that sleep loss would cause the decline of cognition and operation performance. But the effects are complex and it might be related to the factors such as the type of cognitive and operation work. In addition, sleep deprivation also affects mood state and the secretion of some hormones such as cortisol, thus bringing potential hazard to health. Therefore, sleep disturbance is an important risk factor in medium and long term manned spaceflight, especially in interstellar travel. Next, researches about the effects of sleep on immune function, hormone secretion, gastrointestinal function and cardiovascular health should be conducted; further study about the effects of sleep disturbance on behavioral performance and group dynamics on-orbit need to be carried out.

The countermeasures against sleep problems in spaceflight include improving sleep environment, designing reasonable on-orbit work-rest schedule, pharmacologic interventions, light treatment, psychological support, crew selection and training, TCM and so on. These measures are often rationally integrated according to the mission characteristics and the practical resource allocation ability. Although some countermeasures have been used on orbit, the effects and the mechanism still need to be further researched. Among them, drug screening and the development of new preparations should be attached with special importance. As a new technology, light treatment is being used and tested on ISS, which should also be considered in the design and construction of Chinese space station. Specific selection and training of crew, especially gene selection, as a potential method, needs to be further tested and verified. Some promising measures such as TCM and transcranial magnetic/electric stimulation have not yet been applied in flight. More ground-based researches and preparations should be carried out, and the on-orbit tests need to be conducted as soon as possible.

## Conclusions

The research on sleep problems is becoming a new frontier of aerospace medicine. The effects of spaceflight on sleep and the countermeasures need to be further studied. The research achievements in this field will not only ensure the safety, health and performance of astronauts in space, but also benefit people living on earth.

## Abbreviations

fMRI: Functional nuclear magnetic resonance imaging
ISS: International space station
NASA: National Aeronautics and Space Administration
POMS: Profile of Mood States Scale
REM: Rapid eye movement
SSLA: Solid-state lighting assembly
SWS: Slow-wave sleep
TCM: Traditional Chinese Medicine
